# The therapeutic concordance approach reduces adverse drug reactions in patients with resistant hypertension

**DOI:** 10.3389/fcvm.2023.1137706

**Published:** 2023-05-05

**Authors:** Valentina Trimarco, Maria Virginia Manzi, Raffaele Izzo, Pasquale Mone, Maria Lembo, Daniela Pacella, Giovanni Esposito, Angela Falco, Carmine Morisco, Paola Gallo, Gaetano Santulli, Bruno Trimarco

**Affiliations:** ^1^ Department of Neuroscience, Reproductive Sciences, and Dentistry, “*Federico II*” University, Naples, Italy; ^2^ Department of Advanced Biomedical Sciences,“*Federico II*” University, Naples, Italy; ^3^ Department of Medicine, Wilf Family Cardiovascular Research Institute, Einstein-Mount Sinai Diabetes Research Center (*ES-DRC*), Albert Einstein College of Medicine, New York, NY, USA; ^4^ International Translational Research and Medical Education (*ITME*) Consortium, Academic Research Unit, Naples, Italy; ^5^ Department of Public Health, “*Federico II*” University, Naples, Italy; ^6^ Department of Molecular Pharmacology, Fleischer Institute for Diabetes and Metabolism (*FIDAM*), Einstein Institute for Aging Research, Einstein Institute for Neuroimmunology and Inflammation (*INI*), Albert Einstein College of Medicine, New York, NY, USA

**Keywords:** adherence, adverse drug reactions, blood pressure, compliance, concordance, hypertension, pharmacologic resistance, resistant hypertension

## Abstract

**Background:**

Adverse drug reactions (ADRs) remain among the leading causes of therapy-resistant hypertension (TRH) and uncontrolled blood pressure (BP). We have recently reported beneficial results in BP control in patients with TRH adopting an innovative approach, defined as therapeutic concordance, in which trained physicians and pharmacists reach a concordance with patients to make them more involved in the therapeutic decision-making process.

**Methods:**

The main scope of this study was to investigate whether the therapeutic concordance approach could lead to a reduction in ADR occurrence in TRH patients. The study was performed in a large population of hypertensive subjects of the Campania Salute Network in Italy (ClinicalTrials.gov Identifier: NCT02211365).

**Results:**

We enrolled 4,943 patients who were firstly followed-up for 77.64 ± 34.44 months, allowing us to identify 564 subjects with TRH. Then, 282 of these patients agreed to participate in an investigation to test the impact of the therapeutic concordance approach on ADRs. At the end of this investigation, which had a follow-up of 91.91 ± 54.7 months, 213 patients (75.5%) remained uncontrolled while 69 patients (24.5%, *p* < 0.0001) reached an optimal BP control. Strikingly, during the first follow-up, patients had complained of a total of 194 ADRs, with an occurrence rate of 68.1% and the therapeutic concordance approach significantly reduced ADRs to 72 (25.5%).

**Conclusion:**

Our findings indicate that the therapeutic concordance approach significantly reduces ADRs in TRH patients.

## Introduction

While health care systems are known to be highly incentivized to provide care for acute illnesses such as myocardial infarction and stroke, they are not well designed for preventive care (1–3). Primary prevention of cardiovascular disease may potentially be better managed in a system non-centered on the acute care model (4–6). Hence, it is time to acknowledge that standard strategies for blood pressure (BP) management in clinical practice are failing to achieve ideal BP control rates (7–11). New approaches are needed, ideally requiring multifaceted personalized solutions tailored to the needs of specific patients, communities, and health care professionals (12, 13).

Therapy-resistant hypertension (TRH) is defined by BP targets not achieved despite treatment with at least 3 anti-hypertensive drugs of different classes, including a blocker of the renin-angiotensin system, a diuretic, and a long-acting Ca^2+^ channel blocker (14, 15). TRH is currently observed in more than 10% of hypertensive patients. We have recently reported (16) the favorable results in BP control obtained in a population of patients with TRH applying a clinical approach in which a trained pharmacist was responsible for evaluating the medical pharmacological history, in order to remove drug-drug negative interactions, and the physicians aimed at reaching a concordance with the patients in order to make them better informed, actively engaged, and somehow more gratified. We have demonstrated that therapeutic concordance allows to reach a satisfactory BP control in 25% of patients with uncontrolled TRH (16).

Substantial evidence indicates that 20%–90% of patients can experience adverse events attributable to their antihypertensive therapies (17–20); hence, adverse drug reactions (ADRs) are listed among the main causes of uncontrolled BP by reducing patients' compliance (18, 21–29). On these grounds, we designed the present study to investigate whether the therapeutic concordance approach could induce a reduction in ADR occurrence. The principles of the concordance are centered on better communication and engaging patients in treatment decisions. Etymology may help understanding the meaning of therapeutic concordance: the word concordance comes from the Latin term “*concordare*”, which means with the same heart, inferring mutual trust and consensus between two parties in the decision-making process.

The approach of concordance that allows patient autonomy was conceived in the UK in 1997, when the *Royal Pharmaceutical Society of Great Britain* published a report entitled “From Compliance to Concordance: Achieving Shared Goals in Medicine Taking” (30), describing the new model of therapeutic concordance, in which patients and doctors work together towards shared therapeutic goals. Initially limited to the consultation process, in which doctor and patient agree on therapeutic decisions that incorporate their respective views, the definition of therapeutic concordance also includes prescribing communication and patient support in medicine taking. Concordance should not be confused with compliance or adherence (31–33). Indeed, compliance is “the extent to which the patient's behavior matches the prescriber's recommendations”, whilst adherence is defined as “the extent to which the patient's behavior matches agreed recommendations from the prescriber”.

Health-care professionals adapt their style to meet the individual needs of patients and to make information about chronic conditions and their treatment as accessible as possible. They enquire sympathetically about side-effects and ask patients whether they realize the merits of treatment. They counsel patients on the importance of concordance and how to organize their medication, enlisting the help of family members and reiterate the potential risk of sudden unauthorized withdrawal of medications.

## Subjects and methods

### Study design and participants

The study was performed in a population of hypertensive patients of the Campania Salute Network (CSN), which is an open electronic registry [currently including more than 15,000 hypertensive subjects (34)] that fosters the interaction between the Hypertension Research Center of “Federico II” University Hospital in Naples and primary physicians as well as hypertension clinics within the Campania region in Italy (ClinicalTrials.gov: NCT02211365) (34–37).

Patients with uncontrolled office BP (OBP ≥ 140/90 mmHg) and home BP (HBP > 135/85 mmHg), were included in a long-term follow-up. At the end of this procedure, the patients that despite taking 3 antihypertensive drugs including a diuretic, showed uncontrolled office BP and HBP were included in the arm of true TRH (tTRH); instead, patients with uncontrolled office BP but with optimal HBP (white coat effect) were entered in the arm of apparent (aTRH) (38, 39).

Thereafter, tTRH patients were included in the intervention arm of therapeutic concordance, with follow-up visits scheduled at 6-month intervals. Details about the therapeutic concordance approach have been previously described (16).

### Cardiovascular risk factors and disease assessment

Demographics, relevant risk factors, and main clinical characteristics were acquired at enrollment, including age, race, sex, smoking status, weight, diabetes, history of stroke and heart attack history.

For each patient, the physician registered the types of antihypertensive drugs, their dosage, and the use of other drugs alongside with information about the actual length of the anti-hypertensive treatment and the adverse events spontaneously reported by the patients. The term ADRs implies a causal relationship with the anti-hypertensive treatment; so, ADRs were confirmed using the Liverpool Algorithm (40), consisting of the following scale of 4: “Unlikely”, “Possible”, “Probable”, and “Definite”.

Following current guidelines (41), BP was measured after five minutes resting in the sitting position, three times at one-minute interval; all enrolled subjects were also encouraged to measure their HBP utilizing validated devices (41). Follow-up BP and HBP were defined as optimally controlled if the average OBP values was <140/90 mmHg or if the average HBP self-reported value was <135/85 mmHg, respectively (42). The estimated glomerular filtration rate was achieved by means of the Chronic Kidney Disease Epidemiology Collaboration equation, as described (43).

### Study endpoints

Our primary endpoint was to assess whether the use of a therapeutic concordance approach could reduce ADRs. Secondary endpoint was whether a reduction in ADRs, if any, correlates with an improvement in BP control.

### Statistical analysis

Data were analyzed using Jamovi and SPSS (version 25.0; IBM, Armonk, NY) and expressed as mean ± SD. The normal distribution of values was confirmed applying the Shapiro-Wilk test. Continues variables were analyzed using the Student's *t* test. We rejected the null hypothesis at a two-tailed *p* < 0.05.

## Results

From the initial CSN cohort of 5,331 hypertensive subjects with ascertained HBP measurements, and absence of coronary heart disease, we excluded patients with secondary hypertension as well as individuals with a follow-up of less than twelve months, obtaining 4,943 subjects. These patients were firstly monitored for 77.64 + 34.44 months, yielding to the identification of 322 patients with a white coat effect (aTRH, i.e., uncontrolled OBP and optimal HBP) and 4,057 subjects without TRH.

Therefore, from the first follow-up study, we obtained 564 tTRH subjects with uncontrolled OBP (BP ≥ 140/90 mmHg) and HBP (BP > 135/85 mmHg) according to the above-mentioned definition. Since some of these patients did not accept to participate in our study testing the therapeutic concordance approach, we obtained a population of two-hundred eighty-two tTRH patients who in the first follow-up did not display any significant change in BP. The baseline characteristics of these patients are reported in Table 1.

**Table 1:** Baseline characteristics of patients with true therapy-resistant hypertension enrolled in the study.

Variable	Value
*N*	282
Age, year, mean, SD	55.06 ± 10.30
Female sex, *n* (%)	112 (39.7)
Current or former smoker, *n* (%)	139 (49.3)
Diabetes, *n* (%)	66 (23.4)
SBP, mmHg, mean, SD	150.2 ± 20.4
DBP, mmHg, mean, SD	83.3 ± 10.64
HR, bpm, mean, SD	71.2 ± 11.9
Glycemia, mg/dl, mean, SD	103.2 ± 24.1
Creatinine, mg/dl, mean, SD	1.0. ± 0.2
Uric acid, mg/dl, mean, SD	5.5 ± 1.5
Triglycerides, mg/dl, mean, SD	142.7 ± 75.2
Total cholesterol, mg/dl, mean, SD	207.5 ± 40.2
HDL cholesterol, mg/dl, mean, SD	49.3 ± 12.6
Serum potassium, mg/dl, mean, SD	4.3 ± 0.4
Year of hypertension, mean, SD	8.74 ± 7.93
Number of hypertension drugs, mean, SD	3.57 ± 0.65
Follow-up, year, mean, SD	6.46 ± 2.87

During the first follow-up period, 194 ADRs were recorded (Table 2) with an occurrence rate of 68.8%. Thereafter, the hypertensive subjects were monitored according to the therapeutic concordance protocol for a second follow-up period, which lasted 91.91 + 54.7 months.

**Table 2:** Percentage of adverse reactions recorded before the concordance therapeutic approach. The population has been divided in subgroups according to the BP response during the second follow-up period (Group 1 without, Group II with optimal BP control). Data are expressed as number (%).

Adverse reaction	BP < 140/80 mmHg (Group II, *N* = 69)	BP > 140/80 mmHg (Group I, *N* = 213)
Tachycardia events	0	1 (0.5)
Itch	1 (1.4)	1 (0.5)
Heartburn	1 (1.4)	1 (0.5)
Syncope	1 (1.1)	1 (0.5)
Asthenia	4 (5.8)	6 (2.8)
Cough	5 (7.2)	18 (6.6)
Cramps	3 (4.3)	6 (2.8)
Reduced libido	1 (1.4)	8 (3.8)
Constipation	0	3 (1.4)
Lip edema	0	1 (0.5)
Dyspnea	2 (2.9)	2 (1.9)
Bradycardia	1 (1.4)	1 (0.5)
Erectile dysfunction	2 (2.9)	5 (2.4)
Hypotension	4 (5.8)	12 (5.6)
Hypokalemia	1 (1.4)	4 (1.9)
Gingivalhyperplasia	0	1 (0.9)
Tremor	0	2 (0.5)
Flushing	1 (1.4)	4 (1.9)
Leg edema	714 (20.2)	51 (24.0)
Headache	1 (1.4)	9 (4.2)
Gum Bleeding	0	1 (0.5)
Increased transaminases	0	2 (0.9)
Hematuria	0	1 (0.5)
Polyuria	0	2 (0.9)
Erythema	1 (1.4)	2 (0.9)
Dysgeusia	1 (1.4)	2 (0.9)
Dizziness	2 (2.9)	2 (1.9)

**Figure 1:**
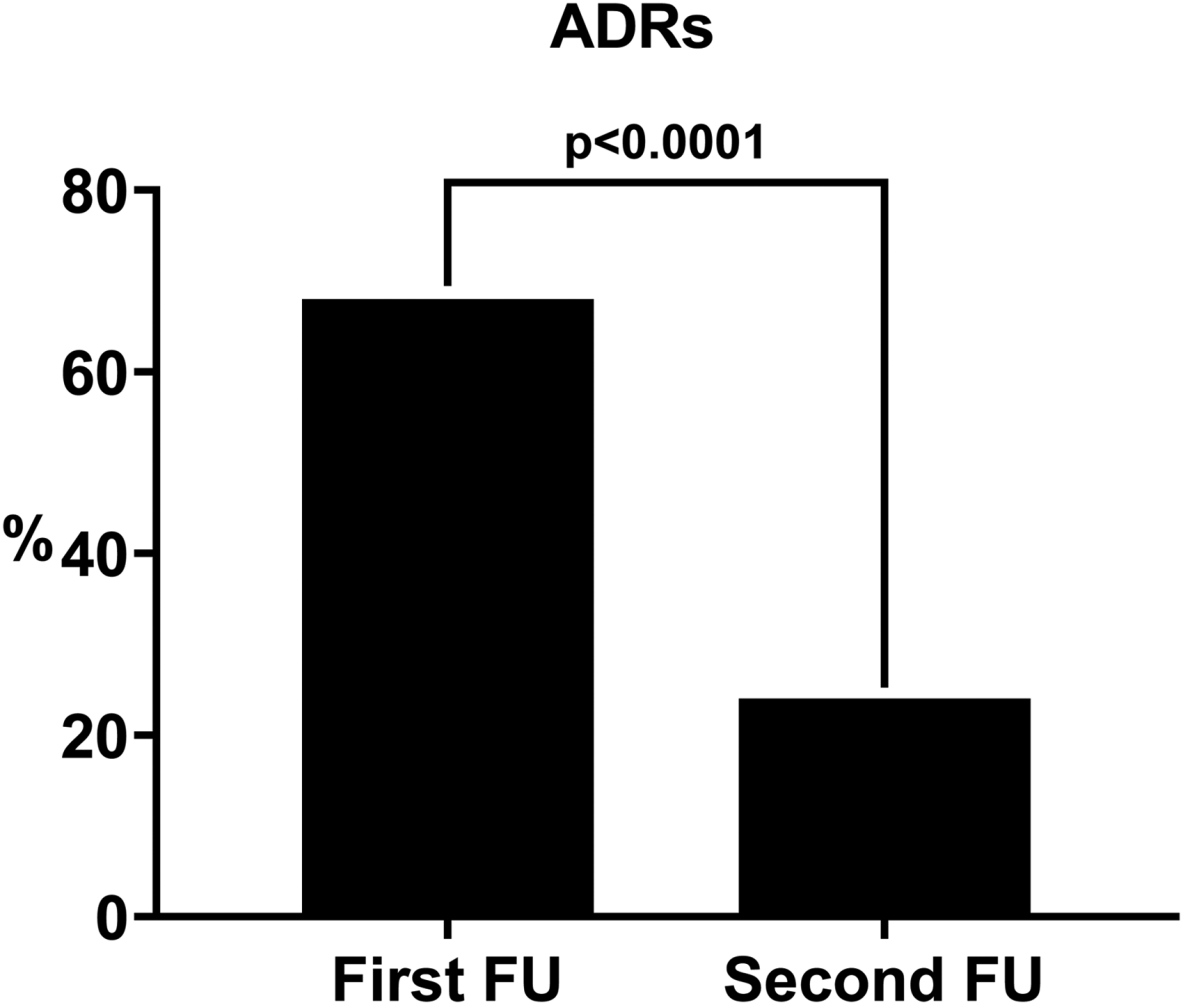
Percentage of patients with ADRs before (first FU) and after (second FU) the therapeutic concordance approach. FU: follow-up.

After the therapeutic concordance approach 213 patients (Group I: 75.5%) remained uncontrolled (uncontrolled tTRH) whereas 69 patients (Group II: 24.5%) were able to reach an optimal BP control (defined as an average BP inferior to 140/90 mmHg in >50% of the follow-up visits). During this second follow-up 72 ADRs were recorded (Table 3), with an occurrence rate of 25.5%, which was statistically lower as compared to the percentage recorded in the first follow-up (Figure 1) despite thefact that there was no statistically significant reduction in the mean daily number of antihypertensive drugs and in the percentage of use of the main classes of antihypertensive medications (Table 4).

**Table 3:** Type of adverse reactions recorded during the follow-up period after therapeutic concordance approach. The population has been divided in subgroups according to the BP response during the second follow-up period (Group 1 without, Group II with optimal BP control). Data are expressed as number (%).

Adverse reaction	BP < 140/80 mmHg (Group II, *N* = 69)	BP > 140/80 mmHg (Group I, *N* = 213)
Tachycardia events	0	2 (0.9)
Itch	1 (1.4)	2 (0.9)
Heartburn	1 (1.4)	
Syncope	1 (1.4)	1 (0.5)
Asthenia	1 (1.4)	
Cough	1 (1.4)	3 (1.4)
Cramps	1 (1.4)	2 (0.9)
Reduced libido	0	1 (0.5)
Hyperuricemia	0	1 (0.5)
Lip edema	0	1 (0.5)
Increasedbloodcreatinine	0	1 (0.5)
Bradycardia	0	1 (0.5)
Erectile dysfunction	0	1 (0.5)
Hypotension	0	4 (1.9)
Hypokalemia	0	2 (0.9)
Gingival hyperplasia	0	1 (0.5)
Tremor	0	1 (0.5)
Flushing	1 (1.4)	2 (0.9)
Leg edema	7 (10.1)	31 (14.8)
Angioedema	0	1 (0.5)

**Table 4:** Antihypertensive drugs used before (first follow-up period) and after (second follow-up period) the application of the therapeutic concordance approach. The population has been divided in subgroups according to the BP response during the second follow-up period (Group I without optimal BP control, Group II with optimal BP control). Statistical analysis was performed comparing the percentage of each class of drug used in the two follow-up periods in the same group of patients, and no significant differences were observed (all *p* values were >0.05).

Drug Class (%)	Follow-up period	(Group I, *N* = 213)	(Group II, *N* = 69)
Calcium Channel Blockers	First	62.7	53.6
	Second	65.9	46.8
Diuretics	First	100	100
	Second	100	100
α Adrenergic Receptor Blockers	First	23.1	21.9
	Second	16.9	14.6
β Adrenergic Receptor Blockers	First	44.8	52.5
	Second	55.2	45.8
ACE-I/ARBs	First	97.2	95.7
	Second	93.4	88.4
Aldosterone Blockers	First	55.3	51.5
	Second	59.1	47.2

ACE-I, angiotensin converting enzyme inhibitors; ARB, angiotensin receptor blockers (sartans).

A statistically significant difference in the rate of occurrence of ADRs was detected between males and females (rate of ADR occurrence 20.5% vs. 30.3%, respectively, *p* = 0.037), such a difference was not seen in the first follow-up (females 73%, males 65.8%. n.s.). Further, we divided the patients into four age quartiles (<47.7 years; 47.8–55.1 years; 55.2–61.9 years; >70 years) and we observed that ADRs due to antihypertensive treatments were more common in third age quartile: ADRs in this age group were 26 (37.5%). Individuals who developed ADRs in the other age groups were 11 (15.7%), 19 (26.8%), and 16 (22.5%) in the first, second, and fourth quartiles, respectively (*p* for trend < 0.05). On the contrary, during the first follow-up period we did not detect any significant difference in the rate of ADRs occurrence between the four age groups: 46 (65,7%), 54 (76,0%), 53 73,6%) and 41 (57,7%), respectively from the first to the fourth quartile).

**Figure 2:**
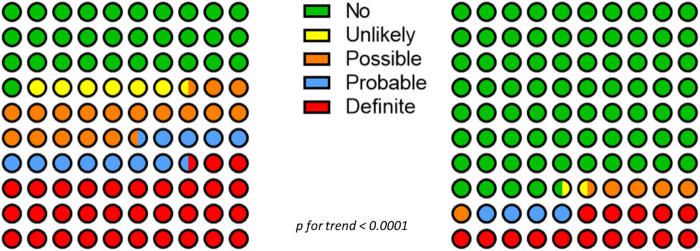
Distribution of ADRs before (first follow-up, right panel) and after (second follow-up, left panel) therapeutic concordance according to the Liverpool algorithm.

According to the Liverpool scale, 3 (4.1%) ADRs recorded during the second follow-up were “Unlikely”, 16 (22.2%) “Possible”, 10 (13.9%) “Probable”, and 43 (59,7%) were “Definite”, a distribution which was not different from that observed during the first follow-up when 18 (9.3%) were “Unlikely”, 50 (25,8%) “Possible”, 34 (17.5%) “Probable” and 92 (47.4%) “Definite” (Figure 2).

During both follow-up periods no serious ADRs were recorded. Of the 282 patients who successfully completed the second follow-up study, 69 obtained a satisfactory BP control with 2.9 + 1.0 antihypertensive drugs/day, 27 (9.57%) reached BP control with <3 antihypertensive drugs (including a full dose diuretic), while 42 (14.9%) patients required more than 3 antihypertensive drugs and were thereby considered patients with difficult-to-control tTRH. The remaining 212 patients (75.5%) were tTRH since they failed to obtain a satisfactory BP control despite a greater daily number of antihypertensive drugs 4.25 + 1.0 (*p* < 0.01 vs. 2.9 + 1.04).

However, no significant difference in ADR occurrence rate was detected between Group I (*n* = 210) and Group II (*n* = 69) (25.8% vs. 20.3%, *p* < 0.353), notwithstanding the lower number of antihypertensive drugs in Group II.

## Discussion

In the present study we investigated whether an evaluation of the pharmacological history of the patient and a more empathetic approach that actively involves the patient in the choice of her/his antihypertensive treatment could improve the tolerability of the antihypertensive therapy.

Our findings indicate a significant reduction in the rate of occurrence of ADRs, which in about 25% of the population of patients defined as tTHR hypertensives is associated with an improved BP control, so that they were no longer defined as resistant to the treatment or at least became difficult to control hypertensives, who required more than three drugs to achieve a good BP control. Howbeit, a comparable reduction in the rate of ADR occurrence was recorded in the remaining 3/4 of our population who failed to obtain any improvement in BP control, so it seems likely that the reduction in ADR occurrence improves BP control but not vice versa.

Although physician-related barriers for concordance have been considerably minimized by the introduction of clearer guidelines, physicians may not be sufficiently insistent in the management of hypertension, especially in older adults (44–49). In this regard, it is important to emphasize that most of the side effects declared by the patients have no relationship with antihypertensive drugs, while other adverse effects, including reduced libido and hypokalemia have a reduced incidence as compared to the one expected, probably on account of a correct use of drug combination.

Since all patients received more than one single drug, our findings do not consent speculations on the tolerability of single antihypertensive drugs, albeit some ADRs are considered specific for one class of drugs, such as leg edema for calcium channel blockers (50). Finally, although we did not measure adherence and persistence of the prescribed treatment, the finding that a reduction in ADRs is accompanied by an improvement in BP control allows the speculation that the presence of ADRs may impair these two parameters. Nevertheless, since no difference in ADR rate occurrence could be detected between patients with and without a satisfactory BP control, ADRs cannot be considered the only responsible of unsatisfactory BP control by reducing the adherence to the antihypertensive regimen, at least when the pharmacologic prescription has taken into account possible drug to drug interactions and has been agreed with the patient, as in the concordance protocol. The usefulness of this global approach is further demonstrated by the observation that in a population requiring a large number of antihypertensive drugs we have observed a very low rate of ADR occurrence so that we have been able to detect some characteristics of ADR occurrence, such as the higher rate of ADRs in females and in subjects ageing 55–62 years, which seem to be peculiar of antihypertensive monotherapy.

The major limitation of our study is that it has not been planned and performed according to a double-blind placebo-controlled protocol. Notwithstanding, the use of two very long follow-up periods and the choice to perform the study in patients with tTRH may allow to overcome this limitation. Furthermore the availability of two very long follow-up periods, each lasting more than 6 years, and the choice to perform the study in patients with tTRH minimize the role of other factors, such as changes in BMI or in physical activity as well progressive familiarization of patients with the staff, in the improvement of antihypertensive treatment tolerability.

## Conclusions

In summary, our data indicate that the therapeutic concordance approach is able to significantly reduce the occurrence of ADRs in tTRH patients. Our findings support a thorough rearrangement of current health care systems in order to emulate rigorous management strategies used in clinical trials proven to provide better BP control rates than traditional approaches.

## Data Availability

The raw data supporting the conclusions of this article will be made available by the first authors, upon reasonable request, without undue reservation.
